# Simple model systems: a challenge for Alzheimer's disease

**DOI:** 10.1186/1742-4933-9-3

**Published:** 2012-04-16

**Authors:** Marta Di Carlo

**Affiliations:** 1 Istituto di Biomedicina ed Immunologia Molecolare (IBIM) Alberto Monroy CNR, via Ugo La Malfa 153, 90146 Palermo, Italy

**Keywords:** Age, Neurodegenerative disease, Animal model, Misfolding, Protein aggregation

## Abstract

The success of biomedical researches has led to improvement in human health and increased life expectancy. An unexpected consequence has been an increase of age-related diseases and, in particular, neurodegenerative diseases. These disorders are generally late onset and exhibit complex pathologies including memory loss, cognitive defects, movement disorders and death. Here, it is described as the use of simple animal models such as worms, fishes, flies, *Ascidians *and sea urchins, have facilitated the understanding of several biochemical mechanisms underlying Alzheimer's disease (AD), one of the most diffuse neurodegenerative pathologies. The discovery of specific genes and proteins associated with AD, and the development of new technologies for the production of transgenic animals, has helped researchers to overcome the lack of natural models. Moreover, simple model systems of AD have been utilized to obtain key information for evaluating potential therapeutic interventions and for testing efficacy of putative neuroprotective compounds.

## Introduction

**Figure 1:**
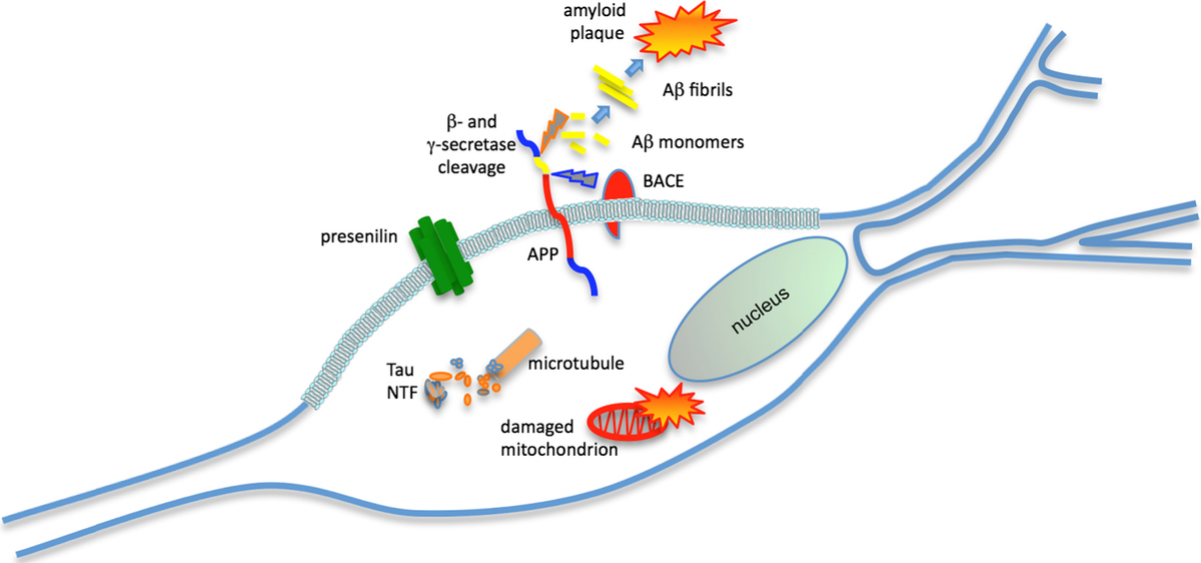
**The two pathological hallmarks of AD are extracellular plaques and intracellular tangles**. Plaques are formed mostly from the deposition of amyloid beta (Ab) a peptide derived from amyloid precursor protein (APP). The metabolic processing of APP that results in Ab formation requires two enzymatic cleavage events, a b-secretase cleavage by the aspartyl protease beta-site APP-cleaving enzyme (BACE) and a g-secretase cleavage dependent on presenilin. Single beta-amyloid peptides, after misfolding, can aggregate and form fibrils and successively plaques. Filamentous neurofibrillary tangles (NTF) are formed from paired helical filaments composed of hyperphosphorylated tau protein, a microtubule-associated protein.

Alzheimer's disease (AD) is the most common form of dementia. This incurable, degenerative, and terminal disease is usually diagnosed in people over 65 years of age, although the less-prevalent early-onset AD can occur much earlier. The morphologic features observed in AD patients at autopsy include both extracellular amyloid deposits as amyloid senile plaques and intracellular neurofibrillary tangles (NFT). The main constituent of the amyloid deposits is an amphiphilic peptide, derived by proteolysis from a large membrane spanning precursor protein, the amyloid precursor protein (APP). According to the amyloid hypothesis cascade, the beta-amyloid (Aβ) peptide deposits are the fundamental cause of the disease [1]. Depending on cellular conditions, Aβ is misfolded and the establishment of Aβ conformations, prone to self-assembling, could represent a key point of the neurodegenerative process. The intermolecular aggregation, prompted by instability, strongly correlates to the increase of ordered structures rich of beta-sheets, typical of amyloid assemblies. Fibrillar forms of Aβ found in amyloid plaques were previously considered the major cause of neuronal damage in AD, but recently it has been discovered that the Aβ soluble oligomers, also known as Aβ-derived diffusible ligands (ADDLs), are the more potent neurotoxins [2]. Another AD hallmark are the NFT, composed by neurofilaments and hyperphosphorylated *tau *protein, a microtubule associated polypeptide. These two markers are common both to late-onset/sporadic AD and early-onset/familial AD (FAD) suggesting a common pathogenic pathway [3]. However, it is not yet well clear, if the presence of these two hallmarks is the cause or the effect of a cascade of events including oxidative stress, mitochondrial damage and death for apoptosis. A scheme of these events is showed in Figure 1.

A model organism is a non-human species that is extensively studied to understand particular biological phenomena, with the expectation that discoveries made in the organism model will provide insight enforceable for other and more complex organisms. In particular, model organisms are widely used to explore potential causes and treatments for human disease when human experimentation would be unfeasible or unethical. This strategy is made possible by the common descent of all living organisms, and the conservation of metabolic and developmental pathways and genetic material over the course of evolution. The study of model organisms can be informative, but care must be taken when generalizing from an organism to another one. Moreover, in developing an animal model system for human disease, an appropriate model should have a close evolutionary relationship to humans. Consequently, mice have been used extensively as AD animal models due to the similarity with the human brain anatomy and the existence of numerous behavioral tests to examine neural dysfunction [4]. However, despite the genomic similarities to humans, mouse and other model organisms typically do not contract the same genetic diseases, so scientists must alter their genomes to induce human disease states. In attempting to engineer a genetic mouse model for a human disorder, for example, it is important to know what kind of mutation causes the disease, so that the same kind of mutation can be introduced into the corresponding model organism genome. Scientists approach this task in two main ways: one that is directed and disease driven, and the other one that is undirected and mutation driven. The undirected mutation-driven method uses radiation and chemicals to cause mutations. On the other hand, the directed disease-driven approach can employ any techniques, depending on the exact type of mutation involved in the disease under study. Common directed techniques include transgenesis or single-gene knock-outs and knock-ins. Transgenic animals are generated by adding foreign genetic information to the nucleus of embryonic cells, thereby inhibiting gene expression. This can be achieved by either injecting the foreign DNA directly into the embryo or by using a retroviral vector to insert the transgene into an organism's DNA. Both knock-out and knock-in models are ways to target a mutation to a specific gene locus. These methods are particularly useful if a single gene is shown to be the primary cause of the disease. Knock-out mice carry a gene that has been inactivated, which creates less expression and loss of function; knock-in mice are produced by inserting a transgene into an exact location where it is overexpressed.

**Figure 2:**
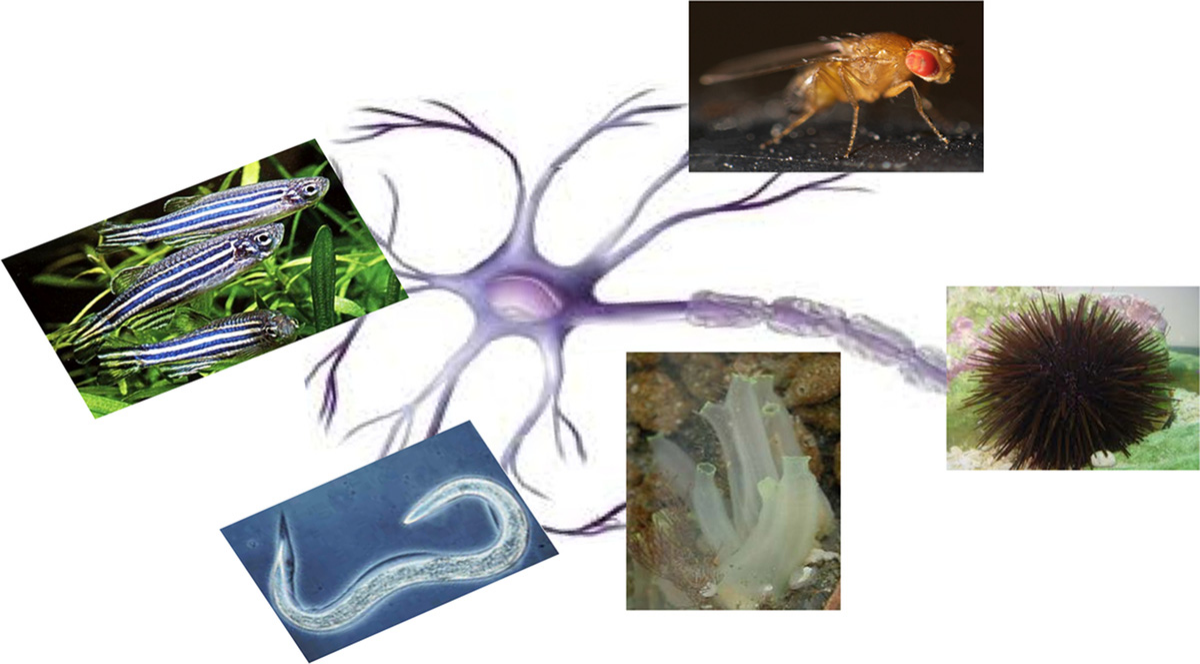
**Simple model systems as zebrafish, Drosophila, *C. elegans*, Ascidian and sea urchin have been used to study neurodegeneration**.

To generate transgenic mice is not only time-intensive but also costly. For these reasons, researchers have turned their attention to invertebrate animal models that have provided much insight into some of the molecular mechanisms involved in AD pathogenesis (Table 1). Modeling human disease in simple invertebrate systems is attractive because genetic screens can be performed in a relative short time to identify mutations leading to age-dependent neurodegeneration. Invertebrate models should give insight into toxic activities of disease-related human proteins that, in every case, need to be validated in mammalian systems. The nematode *Caenorhabditis elegans*, the zebrafish *Danio rerio*, the fruit fly *Drosophila melanogaster*, the Ascidian *Ciona intestinalis *and the *Strongilocentrotus purpuratus *and *Paracentrotus lividus *sea urchins, transgenic or not, can offer many advantages to obtain new knowledge about the toxic mechanisms underlying this human neurodegenerative disease (Figure 2). In addition, these simple model systems have its genome completely sequenced and surprisingly, many of the human disease genes have the counterpart in the genes of these models. Regardless of their obvious simplicity, these models allow to identify protein interactions, useful for understanding the entire regulatory pathways.

**Table 1:** The research application of the most common invertebrate models used for AD

ANIMAL MODEL	COMMON NAME	RESEARCH APPLICATION
*Caenorhabditis elegans*	Roundworm	Localization of amyloid deposit Effect of aging on the size of amyloid plaques Aβ toxicity and behaviour correlation Effect of dietary deprivation in AD

*Danio rerio*	Zebrafish	Studies on NTF and amyloid plaques Toxicity of Aβ peptide Mutations in APP, PSEN1 and PSEN2 genes Drug screening

*Drosophila melanogaster*	Fruit fly	Assessment of modulators of BACE1 or APP metabolism Localization of Aβ peptide Role of *tau *protein in AD

*Ciona intestinalis*	Ascidian	APP processing Study on familial AD Drug screening

*Paracentrotus lividus*	Rock sea urchin	Relationship between different Aβ aggregation forms and toxicity Different activation of Apoptotic pathways

*Sphaerechinus granularis*	Violet sea urchin	Neuroprotective effect of neurotransmitters Developmental abnormalities due to Aβ peptide administration

### A worm as AD model system

The nematode (roundworm) *Caenorhabditis elegans *(*C. elegans*) is a transgenic useful model to study common and fundamental toxic mechanisms underlying human neurodegenerative diseases. *C. elegans *is a free-living nematode about 1mm in length, which lives in temperate soil environments. The worm is transparent, thus facilitating the study of cellular differentiation and other developmental processes in the intact organism, and results one of the simplest organisms with a nervous system [5]. The latter comprises 302 neurons [6,7] and the neuronal classes include chemosensory, mechanosensory and thermosensory. Formation, trafficking, and release of synaptic vesicles in *C. elegans *are highly conserved, employing many of the same proteins used in mammalian neurons.The information available about this organism has permitted to develop transgenic disease-associated human protein models [8]. Concerning neurodegenerative diseases, a relevant advantage of *C. elegans *models is its short lifespan, which allows both rapid construction of different transgenic models and quick assessment of the experimental interventions. Several attempts were done in the past to generate a transgenic AD worm based on the increased production of Aβ [9]. Unfortunately, although the *C. elegans *genome includes genes encoding proteins related to human APP, these genes do not possess the region encoding the neurotoxic Aβ [10]. So, the generation of an AD model by mutation of endogenous APP cleavage was ineffective. However, some researchers developed a transgenic *C. elegans *model able to express the human Aβ fragment inside the muscle cells, with a transgene-induced paralysis phenotype [8,11,12]. A construct, called pCL12, containing the chimeric gene unc-54/Aβ_1-42 _was engineerized. The *C. elegans *unc-54 gene encodes the major myosin heavy chain expressed in the body-wall muscle. Its promoter/enhancer sequences that produce high-level muscle-specific gene expression were assembled with a DNA fragment coding for the human Aβ_1-42 _The minigene was introduced into the nematode by gonad microinjection to produce Ab constitutively expressed in the CL2006 strain of *C. elegans*. The identification of the transgenic nematodes were done with the co-injection of pRF4 plasmid which encodes a mutant collagen gene whose expression leads to an easy recognized Roller phenotype [8]. This mutation causes a rolling movement of the worm instead of the normal synusoidal one. Using this procedure, it was found that the amyloid deposits are located intracellularly and the increased amyloid fibril content in individual worm from mid-larva to adult stage was caused by an increase in the deposit size rather than new deposits [12,13]. Successively, a series of transgenic lines were generated expressing potentially non-amyloid variant forms of Aβ in which single amino acid substitutions (e.g. Leu, Pro) dramatically reduced or blocked amyloid formation but it did not reduce toxicity, suggesting that amyloid aggregates itself are not the really toxic species [11]. Transgenic *C. elegans *was also utilized to study the age-dependence of AD investigating whether in bacterial deprivation, a form of dietary restriction that extends lifespan, Aβ toxicity can be reduced [14]. Dietary restriction confers a general protective effect against toxicity and promotes longevity by a mechanism involving heat shock factor -1 (hsf-1). Moreover, *C. elegans *model was also utilized to associate learning and behavior of the worm with Aβ toxicity [14,15]. Many nematodes modify their behaviors in response to the presence or absence of food. The Enhanced Slowing Response (ESR), an experience-dependent learning behavior, relies on a conserved response to starvation. *C. elegans *behavior was investigated after introduction of the human Aβ gene in the nematode, showing decreased lifespan at 23°C, deficits in odorant preference associative learning behavior and reduced serotonin-stimulated egg laying [16,17].

### *Danio rerio*: from the aquarium to neurodegeneration studies

The zebrafish is a tropical freshwater and a common aquarium fish. It is so named for the five uniform, pigmented, horizontal blue stripes on the body. External development and optical clarity during embryogenesis allows for visual analyses of early developmental processes and genetic analyses. As a vertebrate, the basic organization and divisions of the nervous system are similar to those of others species, including humans. The zebrafish CNS contains specialized neuronal populations of direct relevance for human neurodegenerative diseases, for example, dopaminergic neurons, cerebellar Purkinje cells, oligodendrocytes and astrocytes [18-23]. Its anatomic structure and the presence of orthologous of some human genes [18], permit to use zebrafish as model for studying neurodegenerative diseases. Through careful and creative design of screens, indeed, any developmental or clinically relevant process can be studied, and zebrafish provides a forward genetic approach for assigning function to genes, and positioning them in disease-related pathways. Different techniques were used to generate transgenic zebrafish with the aim to investigate about the two hallmarks of AD: NFT and amyloid plaques, starting from microinjection of linearized plasmids or trasposons, or producing constructs with appropriate cis-acting regulatory elements [24]. Different transgenic zebrafish models have been developed and some of them were used to study *tau *protein. In AD, *tau *is hyperphosphorylated, displaced from its normal association with microtubules and deposited into NFT. Since the degree of NFT amount (presence) is closely correlated with the clinical severity [25,26], *tau *protein results to be an important target for research and drug development. A transgenic model system to study the functional consequences and trafficking patterns in zebrafish neurons of human *tau*, either mutated on site associated with AD or altered at selected post-translational modification sites, has been developed. This model produced a cytoskeleton disruption resembling the NTF in human disease and it could be potentially utilized to dissect a hierarchy of mechanisms in AD.

To the purpose of constructing models for investigating Aβ toxicity, several approaches have been done. Simple incubation of zebrafish embryos in media containing Aβ peptide appears to produce effects on their neural development as monitored by changes in the patterns of neurons if compared with non-transgenic zebrafish. Moreover, Aβ can also induce cell- and embryo-degeneration [27]. It has also been generated a transgenic zebrafish model to facilitate screening for drugs suppressing Aβ toxicity, by expressing the human Aβ 42 amino acid residue form of in the melanophores (corresponding to human melanocytes) constituting the zebrafish's dark surface stripes. The hope was to create a highly visible and, at the same time, viable and fertile phenotype zebrafish larvae. The larvae should then be arrayed in microtiter plates to screen compound libraries for drug acting to reduce Aβ toxicity. To this aim it was used a DNA fragment from the promoter of the microphthalmia-associated transcription factor (mitfa) a crucial gene for melanocyte differentiation in zebrafish [28]. This was coupled to DNA encoding for a secretory signal fused to Aβ Unfortunately, fish bearing this transgene only showed an aberrant pigment phenotype at the advanced age of 16 months, too late for the use in drug screening [29]. Nevertheless, the alteration of the zebrafish pigment pattern could be useful to analyzing the toxic peptide action [29]. Mutations in three genes are known to cause familial AD (FAD). The mutations occur in the genes encoding the APP and presenilin 1 (PSEN1) and 2 (PSEN2) and cause the increased secretion of the pathological Aβ transgenic zebrafish was engineered to analyze presenilin function in the hope to understand the role of mutations of its human orthologous in AD.

Zebrafish PSEN1 sequence and activity were previously analysed and conservation of protein primary structure was noted [30]. Gene transcripts were apparently present in all cells at all examined developmental stages. When zebrafish PSEN1 protein expression was driven at high levels in cultured human HEK293 cells, the zebrafish protein displaced human PSEN1 from γ-secretase complexes, indicating sufficient structural conservation to interact with other complex components. Moreover, zebrafish PSEN1 possesses sufficient primary structural differences from human PSEN1 because its proteolitic action mainly produces the Aβ_40 _form. Moreover, it has also been demonstrated that mutation of one of the two critical catalytic aspartate residues in zebrafish PSEN1 could abolish its γ-secretase activity [30].

### A fly in the forefront of AD research

The *Drosophila melanogaster *is a small, common fly findable near unripe and rotten fruit. Wildtype fruit flies have brick red eyes, are yellow-brown in color, and have transverse black rings across their abdomen and exhibit sexual dimorphism. Thomas Hunt Morgan was the pioneer biologist in *Drosophila* studies in the early 1900's, discovering sex linkage and genetic recombination, which placed the small fly in the forefront of genetic research. More recently the biological similarities between human and *Drosophila* genes have permitted to utilize the fly to exploit the field of neurodegenerative diseases, with a great success [31]. The fly has a brain, containing approximately 200,000 neurons, and like the vertebrate central nervous system, it is composed of a series of functionally specialized substructures as sources of sensory input such as optic and olfactory. The neurons are very similar to their human equivalents in terms of shape, synaptic intercommunications and biochemical signatures. These functional and structural similarities allow constructing fly models of human diseases. These models typically involve transgenic fly expressing a human gene bearing a known dominant mutation or expressing a targeted loss-of-function mutation generated in fly orthologous of these genes [32-38]. Fly models of AD are also available to the community and are now providing new insights into disease mechanisms and assisting in the identification of novel targets for therapy [33]. A particular model of Aβ toxicity has been achieved by creating transgenic flies carrying gal4-driven constructs encoding human APP and human beta-site APP-cleaving enzyme 1 (BACE1) able to generate the Aβ peptide [39]. gal4/upstream activating sequence (UAS) system is usefully used in *D. melanogaster*, to achieve tissue-specific transgene expression [40]. In this system, the yeast GAL4 protein is expressed in particular cells by using selected enhancers or promoters. GAL4 activates a chosen gene upon binding to the UAS. This Ab toxicity relatively complex model is ideal for the assessment of modulators of BACE1 or APP metabolism, but, in some aspects, is less easy to handle than the models in which the Aβ sequence is fused downstream of a secretion signal peptide [41-43]. In these latter models, the expressed Aβ peptide is in part released in the extracellular environment. Another fraction, instead, is accumulated intracellularly and can be correlated with early phenotypes such as locomotor dysfunction and severity. Moreover, immunogold electron microscopy reveals that the Aβ peptides localize in the endoplasmic reticulum (ER), Golgi appartus and lysosomes, but not in the nucleus or mitochondria [44]. This finding suggested that the potentially reversible early phenotypes in AD could be mediated by the intracellular accumulation and aggregation of Aβ. Although studies on Aβ can help to understand one crucial aspect of AD pathogenesis, the investigation on the role of *tau *is also of great importance [45,46]. Fly *tau*-overexpression models have allowed to investigate the role of *tau *in AD. Although wild type human *tau *is neurotoxic when overexpressed in neuronal tissues, the rough eye and longevity phenotypes in *Drosophila *model systems are more severe when AD related variants of *tau *are expressed [47], even when *tau *does not form neurofibrillary tangles [48]. Moreover, flies overexpressing wild-type human *tau *can be induced to form intracellular inclusions resembling neurofibrillary tangles, when glycogen synthase kinase 3β (GSK3β) activity is increased [49]. This finding is in agreement with the known human pathways of *tau *toxicity that seems to require hyperphosphorylation of *tau *to speed up the aggregation process. Thus, *Drosophila* models have permitted to obtain new information about the mechanism of the two principal AD hallmarks.

***Ascidian: an urochordata sister of vertebrate***. 

Ascidian is an urochordata (sea squirt), a tunicate widely distributed in different seas. *Ciona intestinalis *is a solitary tunicate with a cylindrical, gelatinous body, up to 14 cm long, covered by a tunic, made of the polysaccharide tunicin. One of the ends of the body is always fixed to rock, coral or solid surfaces. The other end, opposite to the part secured to the substratum, has two openings, the buccal and atrial siphons. *Ciona intestinalis *has attracted the interest of biologists for developmental studies and its mitochondrial and nuclear genomes have been sequenced [50]. Because of their distant evolutionary relationship to vertebrates, it can be difficult to extrapolate findings obtained in non-chordate invertebrates to study human neurological diseases. In contrast to other invertebrate models, *Ascidians*, like humans, are chordates, share a larval notochord, and undergo neurulation to form a dorsal hollow neural tube. Thus, they can be considered the true sister group of vertebrates and provide an excellent genomic background for modeling human diseases [51]. *Ascidians *have a peripheral nervous systems composed of approximately 350 neuronal cells, including an anterior sensory vesicle and a visceral ganglion containing moto-neurons. This simple chordate nervous system is important in coordinating several aspects of the tadpole behavior, including the larval swimming and the ability to respond to the environmental cues, necessary for settlement [52]. To determine whether the ascidian *Ciona intestinalis *could be used as a model for AD, transgenic larvae were generated expressing wild type and mutant forms of human APP (hAPP695). Expression of hAPP695 appears to be processed in a similar way to the well-characterized Aβ cascade. Furthermore, Aβ forms deposits as assessed by thioflavin S, a dye commonly used for staining amyloid plaques [53]. Increased plaques formation can be achieved by introducing point mutations associated with familial AD into hAPP695. Transgenic *Ascidians *expressing Aβ_1-42 _in the larval nervous system display severe deficiencies with their ability to fixing up, a behavioral response that is important for metamorphosis. Moreover, treatment of transgenic larvae with 3-amino-1-propanesulfonic acid (3-APS), an anti-amyloid therapeutic drug, leads both to a decrease in plaque formations, in a dose-dependent manner, and an improvement in larval attachment. Practically, this AD Ascidian model can be employed to identify factors modulating amyloid deposition, the associated disruption of normal cellular function and behaviors [54].

### Sea urchin a model system to correlate different Aβ aggregates and toxicity

Sea urchin is a useful model system for studying problems in early animal development, and more recently it has been used for identifying specific pathways involved in human pathology or as an indicative tool for pharmacological evaluation. Historically, sea urchin has been a key system in elucidating a variety of classic developmental problems and the regulation of early embryo differentiation [55]. Moreover, sea urchin occupies a key phylogenetic position because it is the only nonchordate belonging to Deuterostomes and the results obtained on this embryo can be extrapolated and compared to those of higher eukaryotes such as mammalians. Nervous systems begin to be present with some neurons and neurites in the structure called ciliary band, in the esophagus and intestine [56]. All the morphological and biochemical events that appear perfectly synchronous in the sea urchin embryo cultures are perturbed when they are exposed to toxic agents of different nature such as metals or teratogens and neurotoxicants and their adverse effects produce uniform phenotypes for a given toxicant and critical exposure period [57]. Furthermore, several clusters of neurons with associated neuropil are organized in ganglia, the largest of which is the apical organ of the larva, composed of 4-6 bilaterally positioned sensory cells containing serotonin. Moreover, *Strongylocentrotus purpuratus *sea urchin genome has been sequenced and despite having no eyes, nose, or ears, has genes involved in vision, hearing and smell in the humans [58]. Moreover, mechanisms that are involved in normal or altered cell homeostasis common to humans have been identified; apoptosis, for example, a mechanism at the basis of neurodegeneration, is well conserved and studied in sea urchin [59,60]. *Paracentrotus lividus *sea urchin embryo has permitted to study the structure-activity relationship between different Aβ aggregation forms and toxicity [61]. Using a recombinant Aβ42 (rAβ42), a preliminary biophysical work has been done to produce rAβ42 different aggregation forms. Small oligomers were obtained at physiological pH, instead larger aggregates at low pH; their size was verified by dynamic light scattering measurements. When sea urchin cultures were incubated with the two different Aβ aggregation species, it was observed that Aβ oligomeric forms significantly increased the level of toxicity with respect to the larger aggregate forms, indicating that the state of Aβ assembly appears to influence their biological activities.

An antigen related to the human APP, called *Pl*APP was identified in sea urchin embryo [62]. *Pl*APP, as in humans, is processed producing a polypeptide of about 10 kDa, suggesting that some molecules and pathways involved in the degenerative process could be conserved during evolution. Using sea urchin it has been possible to find a correlation between Aβ aggregation forms and different apoptotic pathway activations [63]. Aβ aggregates, indeed, induce apoptosis by extrinsic pathway activation, whereas oligomers induce apoptosis both by extrinsic and intrinsic pathway activation. The involvement of mitochondria, pivotal organelles in controlling cell life and death, can explain the major toxicity of oligomers if compared to larger aggregates [63].

Using *Sphaerechinus granularis*, another sea urchin species, it was examined the critical periods in which different types of anomalies are evoked by Aβ and, importantly, established the role played by acetylcholine (ACh) and other neurotransmitters such as serotonin (5HT) and cannabinoids as potential protectants [64]. These morphological studies allow determining the biochemical and molecular mechanisms involved both in the damage caused by the amyloid structures and in the protection provided by the neurotransmitters.

*Sphaerechinus granularis *embryo was also employed to compare developmental abnormalities caused by administration of exogenous APP96-110 and Aβ or different compounds [65]. Although both peptides elicited dysmorphogenesis, Aβ was far more potent; in addition, whereas Aβ produced abnormalities at developmental stages ranging from early cleavage divisions to the late pluteus, APP96-110 effects were restricted to the intermediate, mid-blastula, stage. For both agents, anomalies were prevented or reduced by addition of lipid-permeable analogous of acetylcholine, serotonin or cannabinoids; physostigmine, a carbamate-derived cholinesterase inhibitor, was also effective. In contrast, agents acting on NMDA receptors (memantine) or α-adrenergic receptors (nicergoline), therapeutic in AD, were themselves embryotoxic, as was tacrine, a cholinesterase inhibitor belonging to a chemical class different from physostigmine. Agents acting downstream from receptor-mediated events also provided protection: both the increase of cyclic AMP with caffeine or isobutylmethylxanthine or the administration of the antioxidant α-tocopherol, are all partially effective.

## Conclusions

*C. elegans*, *zebrafish*, *Drosophila*, *Ascidian*, sea urchin, together with other non human model systems no discussed here, summarize some features of AD disease. They have been and will be useful to elucidate mechanistic details and provide the basis for evaluating drug therapies. The simple model systems could allow assessing the pathological importance of a large number of possible modifier genes particularly where orthologous exist. Genes that are found to have a functional importance in simple model systems, as well as showing linkage to AD in humans, will be of particular interest for future detailed studies. Moreover, fundamentally important gene products will be the targets for a new generation of therapeutic compounds for the treatment, or even prevention, of AD. Genetic screens, indeed, can help to define new neuroprotective pathways.

## Abbreviations

AD: Alzheimer's disease; ADDLs: Aβ-derived diffusible ligands; APP: amyloid precursor protein; Aβ: beta-amyloid; BACE1: beta-site APP-cleaving enzyme 1; CNS: central nervous system; ESR: enhanced slowing response; FAD: familial AD; GSK3β: glycogen synthase kinase 3β; hsf-1: heat shock factor -1; NFT: neurofibrillary tangles; PSEN1: presenilin 1; PSEN2: presenilin 2.
